# Children’s Perception of Animacy: Social Attributions to Moving Figures

**DOI:** 10.1177/03010066211010142

**Published:** 2021-05-06

**Authors:** Ruth Hofrichter, Megan E. Mueller, M. D. Rutherford

**Affiliations:** McMaster University, Canada

**Keywords:** animacy, social attribution, social attention, motion perception

## Abstract

Adults describe abstract shapes moving in a goal-directed manner using animate terms. This study tested which variables affect school-aged children’s descriptions of moving geometrical shapes. Children aged 5 to 9 years were shown displays of interacting geometrical shapes and were asked to describe them. Across participants, instructions, number of moving figures, whether a figure caught another, and complexity of the scene were manipulated. Nine-year-olds used significantly more animate phrases than 5-year-olds. Furthermore, we found an Age by Condition interaction. Five-year-olds made significantly more animate statements in the animate condition, while 7-year-olds and 9-year-olds were less affected by instructions. Scene complexity increased children’s use of animate phrases. Number of agents present on the screen and whether a catch occurred did not impact children’s animate attributions. Our results support the hypothesis that children, like adults, are attuned to animacy cues and describe chasing agents in animate terms.

The ability to detect animacy and identify social partners is the first step in social cognition (Szego & Rutherford, 2007). Humans selectively attend to animate objects (New et al., 2007). Even when observing simple geometric shapes moving in a goal-directed manner, people interpret those shapes as having motivations (Heider & Simmel, 1944).

Both children and adults use motion cues to detect animacy (Frankenhuis et al., 2013; Scholl & Gao, 2013). If an object moves against the force of gravity, it is more likely to be judged as animate (Szego & Rutherford, 2008). Agents that accelerate and appear to be self-propelled are also perceived to be animate (Frankenhuis et al., 2013; Pratt et al., 2010). Even when an object does not accelerate, faster moving objects are more likely to be judged as being animate than slower moving objects (Szego & Rutherford, 2007). Furthermore, changing direction or speed, while in motion, are cues to animacy as the ability to deliberately change direction or speed requires agency (Pratt et al., 2010; Szego & Rutherford, 2008; Tremoulet & Feldman, 2000).

Chasing motion is a particularly strong indicator for animacy as it combines multiple motion cues: self-propulsion, acceleration, change in direction, and heat-seeking. Heat-seeking occurs when an agent directly pursues its target (Frankenhuis et al., 2013; Gao et al., 2010). Spatial proximity, the distance between the chaser and its targets, aids in chasing and animacy detection. Closer spatial proximity leads to shorter detection latencies (Meyerhoff et al., 2014). Animacy detection seems irresistible. When adults are shown geometric shapes moving in an animate matter and asked to describe their movement without the use of anthropomorphic terms, they struggle to do so (Heider & Simmel, 1944).

## Children’s Perception of and Preference for Animate Motion

Although animacy detection has evolutionary significance, it is unsurprising that it develops early in life. Newborn infants already show selective attention toward cues that suggest animacy or physical causality (Di Giorgio et al., 2017; Mascalzoni et al., 2013; Valenza et al., 1996). Likewise, newborn animals, like chicks, perceive animacy. Like humans, they prefer to looking at animate agents compared to inanimate motion (Lorenzi et al., 2017; Rosa-Salva et al., 2018). Infants are particularly drawn to chasing motion and display greater pupil dilation when observing a chase between two objects rather than two objects moving randomly (Frankenhuis et al., 2013). By the age of 8 to 10 months, infants do not only recognize a chase but they can also distinguish between the chaser and its target (Rochat et al., 2004). When infants watch a chase, they expect that the chaser will eventually catch its target (Wagner & Carey, 2005).

## Children’s Understanding of Animacy

Three-year-old children know that humans are animate agents with animate characteristics like the ability to initiate action, think and feel things, and experience bodily sensations (Mikropoulos et al., 2003). However, between the ages of 3 and 4, children also regularly attribute animate characteristics to computers and robots (Mikropoulos et al., 2003; Subiaul et al., 2011). By the age of 5, they have a more adult-like understanding of the animate–inanimate distinction and understand that computers and robots are not alive (Mikropoulos et al., 2003). When children ages 5 to 9 years are shown both moving objects and stationary objects, they are more likely to make animate attributions to the moving objects (Poulin-Dubois & Heroux, 1994).

Although many previous studies suggest that children are sensitive to animacy from infancy onwards and perceive moving objects to be animate, Hu et al. (2010) found that young children between the ages of 6 and 9 years do not readily attribute social meaning to cartoon characters in motion. Hu et al. showed children cartoon characters moving around a screen that appeared to be interacting. Participants were asked to describe the displays. Descriptions of younger children tended to focus on characteristics like size and shape of the characters, rather than their actions. Children who were older than 9 years gave more complex responses and made animate attributions. Their responses were focused on how the characters were interacting instead of the characters’ physical characteristics (Hu et al., 2010).

However, while most previous studies tested children’s animate attributions by asking yes or no questions (Mikropoulos et al., 2003; Poulin-Dubois & Heroux, 1994), Hu et al. asked children to describe moving objects in their own words. Giving verbal descriptions might be more challenging for children and impact how many animate attributions they make. Although infants already show attunement toward animacy (Rochat et al., 1997), it is possible that children in Hu et al.’s study recognized the animate nature of the videos but focused on other factors in their verbal descriptions.

## Current Study

This study was designed to investigate how school-aged children (5, 7, and 9 years old) describe geometric shapes that are moving and interacting with one another. Hu et al. (2010) found that children aged 6 to 9 do not readily use animate terms to describe the interactive motion of objects, contrary to previous studies. Therefore, we aimed to test which variables impact children’s verbal descriptions of moving objects. Using a similar technique to Heider and Simmel (1944), children were shown geometrical shapes moving around on a screen. Across trials, two or three shapes were shown engaging in a chase. We chose chasing motion because it carries evolutionary significance (Szego & Rutherford, 2007), and previous studies have shown that both adults and children attend to chasing (Frankenhuis et al., 2013; Rochat et al., 1997). In Hu et al.’s (2010) study, three agents were present throughout the video clips. We varied the number of agents across trials to test if simplifying the interaction might make it easier for children to understand.

Videos used by Hu et al. also showed agents moving in and out of a rectangle shape, representing a house. We manipulated complexity of the videos by including a rectangle shape in half of our videos and only showing the agents in the other videos. Again, we wanted to test if simplifying the videos might affect children’s descriptions of them.

We also included outcome as a variable. Across trials, the chase either ended in a catch or stopped before the chaser could reach its target. Previous research suggested that infants expect a chaser to eventually catch its target; therefore, we wanted to test if outcome would affect children’s descriptions (Wagner & Carey, 2005).

Prior to seeing the clip on each trial, children were given either animate or neutral instructions. Depending on the condition, children were either asked to describe what they had seen or what the dots were doing. The latter instructions were meant to invoke the perception of animacy.

We hypothesized that, across conditions, children would use a greater proportion animate terms in comparison to inanimate terms to describe what they observed. This would stand in contrast to Hu et al.’s (2010) findings. We anticipated that this pattern would be particularly strong in the animate condition, where children were asked what the dots were doing. Based on previous research (Hu et al., 2010; Mikropoulos et al., 2003; Poulin-Dubois & Heroux, 1994), we also expected age differences in how children describe the displays.

## Methods

### Participants

There were three different age groups of participants; twenty-five 5-year-old children (12 females and 13 males; *M *=* *5.305 years, standard deviation [*SD*] = 0.334), twenty-four 7-year-old children (14 females and 10 males; *M *=* *7.310 years, *SD* = 0.313), and twenty-five 9-year-old children (15 females and 10 males; *M *=* *9.106, *SD* = 0.125). Data from one 5-year-old and one 9-year-old participant were excluded due to the children not completing the trials properly. Participants were recruited through an existing research database at McMaster University. Parents and their children were compensated for their time and travel with $10.00. A power analysis based on the standard deviation of Hu et al. (2010) scores (*d *=* *.689) showed that 26 participants were needed per condition (animate vs. inanimate) to be 95% confident that the sample mean would be within the desired margin of error of the true population mean (power = 0.8, α = .05).

### Stimuli

**Figure 1.**
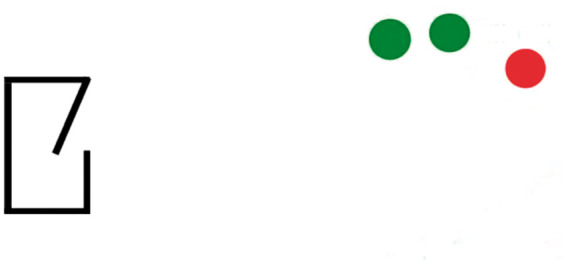
Stimuli display. Two left green dot(s): target(s); Right red dot: chaser.*Note.* Please refer to the online version of the article to view the figure in colour.

The stimuli consisted of a red circle (RGB color = 255,0,0, diameter = 0.17 in.), one or two green circles (RGB color = 5,128,3, diameter = 0.17 in.), and a black rectangular figure (RGB color = 29,29,29, length = 1.4 in., width = 0.8 in.), on a 6.90 in. × 8.62 in. white background (RGB color = 255,255,255). The circles were shown moving around the screen, while the rectangular box, representing a house, stayed stationary. One side of the box would open and close as if it were a door (see Figure 1).

Videos were created in NCH Express Animate Software (for experimental videos and the data set, see https://dataverse.harvard.edu/dataverse/SocialAttributionProject/). In each of the eight clips, the red dot always acted as the chaser and followed the green dot(s) around the screen, directly pursuing them in a heat-seeking manner. The red dot and green dot(s) started each clip approximately 0.5 in. apart. The dots initially traveled at the same speed (for 2.5 seconds), but the red dot quickly sped up to try and catch up to the green dot(s). When the red dot got within close proximity of a green dot (0.2 in.), the green dot would accelerate and change direction to evade the chaser (see Figure 1 for example of stimuli display and Appendix A for video narratives). Across video clips, within-subjects independent variables were number of agents (one or two green dots), complexity (house figure or no house), and outcome (red dot does or does not catch the green dot(s)). Each participant saw all eight video clips. Before running the study, video clips were shown to five adults to confirm that intended and perceived narratives matched and that adults perceived the videos to be animate.

### Procedure

Participants were seated at a table, sitting across from the experimenter. As a warm up task, the experimenter would play a game of Connect Four with the participant. This was meant to give the child a chance to interact with the experimenter and get comfortable with them. Once the child seemed comfortable, the experimenter moved on to the experimental trials. The videos for the experimental trials were displayed on a 6.90 × 8.62-in. touch screen, which was placed on the table, directly in front of the child. Their eyes were on average 19.68 in. from the screen.

Children were randomly assigned to the neutral or animate instruction condition. Across conditions, children were shown the same eight video clips as described earlier. The order in which video clips were shown was randomized for each child. Before the start of each video, the child was told to watch the video closely. In the neutral condition, the child was told, “You are going to watch a video clip on the screen. Watch it closely and after, I want you to tell me what you saw.” In the animacy condition, the child was told “You are going to watch a video clip on the screen. Watch it closely and after, I want you to tell me what the dots were doing.” After each video clip, the experimenter would reiterate the neutral or animate instructions. Once the child responded, the experimenter would move on to the next video clip. If the child did not answer the question, the experimenter would prompt the child again to elicit a response. This procedure continued until the child had seen all eight of the video clips. The procedure was video recorded.

### Coding

A research assistant would watch the video recordings and transcribe the children’s statements. Only task-relevant comments that described the content of the videos were included. Experimenter instructions were not included in the transcripts to keep coders blind to the condition. Transcripts were coded by two separate individuals. One coder was blind to the condition and had no knowledge about the study and associated hypotheses. The other coder was blind to the condition but was aware of what the study entailed. Coders would count the number of statements a child made for each video and categorize each statement according to the coding scheme (see Appendix B). Inter-rater reliability was assessed using the Kappa Statistic. Minimum required Kappa Statistic was 0.7. If minimum required Kappa Statistic was not reached, coders would compare their assessments and discuss conflicts to come to a mutual agreement. If an agreement could not be reached, the blind coder would override the other coder. The blind coder’s data were used for data analysis, as their ratings were more likely to be objective. Out of 576 ratings, raters originally disagreed on 92 ratings.

Once coding was completed, proportion of animate versus inanimate phrases was calculated for each participant by dividing the number of animate phrases used by the individual by the participant’s total number of phrases.

## Results

We fit a linear mixed model using the proportion of animate phrases as our dependent measure with the between-subject variables Participant Age (3: Five, Seven, Nine) and Condition (2: Neutral, Animate), and the within-subject variables Complexity (2: House, No House), Outcome (2: Catch, No Catch), and Number of Agents (2: Two, Three).

**Figure 2.**
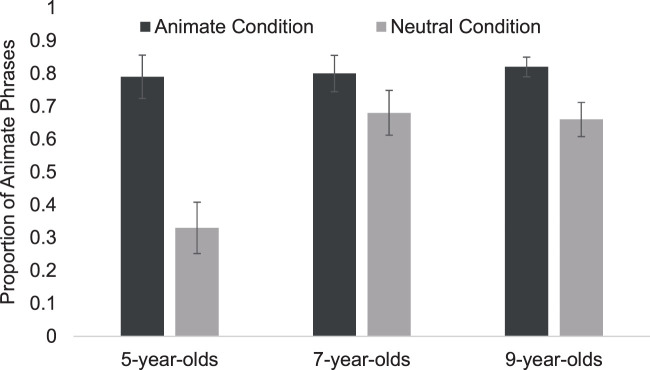
Interaction between participant age and condition. Error bars represent *SEM*.

Results showed a significant main effect of Age, Wald χ^2^ (2) = 9.01, *p *=* *.003. Five-year-olds—*M* = 0.57, *SD* = 0.43, 95% confidence interval [CI] [0.39–0.74]—did not make significantly fewer animate statements than 7-year-olds—*M* = 0.73, *SD* = 0.35, 95% CI [0.59–0.87], *t*(23) = −2.02 *p *=* *.051. However, 5-year-olds (*M* = 0.57, *SD* = 0.43, 95% CI [0.39–0.74]) made significantly fewer animate statements than 9-year-olds (*M* = 0.74, *SD* = 0.30, 95% CI [0.62–0.86], *t*(23) = −2.33, *p *=* *.027. There were no significant differences between the proportion of animate phrases used by 7-year-olds—*M* = 0.73, *SD* = 0.37, 95% CI [0.59–0.87]—compared to 9-year-olds—*M*= 0.74, *SD*= 0.30, 95% CI [0.62–0.86], *t*(23) = −0.18, *p *=* *.85. We found a significant main effect of Condition, Wald χ^2^ (1) = 20.68, *p *<* *.001, in which the proportion of animate phrases used was higher in the Animate Condition (*M* = 0.8, *SD *=* *0.29, 95% CI [0.64–0.96]) than in the Neutral Condition (*M *=* *0.56, *SD *=* *0.39, 95% CI [0.47–0.65], see Figure 2 and Table 1). A significant main effect of Complexity was found, Wald χ^2^ (1) = 19.88, *p *<* *.001, whereby a higher proportion of animate phrases was used in trials that included a house (*M *=* *0.74, *SD *=* *0.33, 95% CI [0.67–0.81]) compared to trials with no house (*M *=* *0.62, *SD *=* *0.40, 95% CI [0.53–0.71]).

**Table 1.** Animate Versus Total Statements Across Age Groups.

	Animate condition							Neutral condition						
	5		7		9			5		7		9		
Age	*M*	*SD*	*M*	*SD*	*M*	*SD*	*M*		*SD*	*M*	*SD*	*M*	*SD*
Animate	11.25	4.55	13.25	6.58	16.91	6.14	4.25		2.63	11.08	5.62	17.17	7.53
Total	15.92	5.74	17.25	6.00	21	7.21	13.42		3.06	18.41	7.82	24.67	7.39

We also observed a significant interaction between Age and Condition Wald χ^2^ (2) = 15.3, *p *<* *.001. Five-year-old children used a higher proportion of animate phrases in the Animate Condition (*M *=* *0.8, *SD*= 0.32, 95% CI [0.66–0.92]) compared to the Neutral Condition—*M* = 0.33, *SD* = 0.4, 95% CI [0.17–0.49], *t*(23)= 4.03, *p *<* *.001, *d *=* *1.27. For 7-year-olds, the proportion of animate phrases used did not differ significantly between the Animate Condition—*M* = 0.79, *SD* = 0.32, 95% CI [0.66–0.92]—compared to the Neutral Condition—*M *=* *0.68, *SD* = 0.37, 95% CI [0.53–0.83], *t*(23) = 0.95, *p *=* *.35, *d *=* *0.35. Likewise, there was no significant difference between the proportion of animate phrases that 9-year-olds used in the Animate Condition—*M* = 0.82, *SD* = 0.26, 95% CI [0.72–0.92]—compared to the Neutral Condition—*M *=* *0.66, *SD *=* *0.33, 95% CI [0.47–0.73], *t*(23)= 0.97, *p *=* *.34, *d *=* *0.54 (see Figure 2). We found no significant main effect of Outcome, Wald χ^2^ (1) = .04, *p *=* *.84, or Number of Agents, Wald χ^2^ (1) = 1.61, *p *=* *.2.

We fit a second linear model using total number of statements with the between-subjects variable Age and the within-subjects variable Condition. There was a main effect of age on the total number of statements made by the child Wald χ^2^ (1) = 17.76, *p *<* *.001 (see Table 1). Five-year-old children—*M* = 14.67, *SD* = 4.68, 95% CI [12.8–16.5]—did not make fewer statements than 7-year olds—*M*= 17.83, *SD*= 6.84, 95% CI [15.1–20.6], *t*(46)= −1.97 *p *=* *.06. However, 5-year-old children (*M* = 14.67, *SD* = 4.68, 95% CI [12.8–16.5]) made fewer statements than 9-year-olds (*M* = 22.91, *SD* = 7.38, 95% CI [20–20.9], *t*(46) = −4.6, *p *<* *.001. Seven-year-olds also—*M* = 17.83, *SD* = 6.84, 95% CI [15.1–20.6]) used fewer statements than 9-year-olds (*M* = 22.91, *SD* = 7.38, 95% CI [20–20.9], *t*(46) = −2.45 *p *=* *.018.

## Discussion

Our results revealed that children in the Animate Condition used a higher proportion of animate statements than those in the Neutral Condition. We expected to see this result because animate instructions imply that the dots are doing something. Prompting children with an animate question might indicate what kind of answer the researcher is expecting. Neutral instructions could be ambiguous, and children may be unsure of what kind of response is expected. When children are primed to interpret dots as animate, their performance is similar to the adults in Heider and Simmel (1944) study who used animate phrases to describe moving geometric shapes.

The age of the participants also had an impact on the proportion of animate statements used, supporting our hypothesis. Previous research has found mixed results regarding the link between age and animate attributions. Poulin-Dubois and Heroux (1994) found that 5- to 9-year-olds made animate attributions when they were presented with displays of moving objects, suggesting that children of this age perceive animacy in such displays and attribute animate characteristics to geometric objects in motion. In contrast, Hu et al. (2010) found that children younger than 9-years-old did not readily make animate attributions and instead focus on shape and size attributes. Younger children (6–9 years of age) only described 27% of social events in the display. Our findings align with Poulin-Dubois and Heroux (1994) as, despite the increase of animate attributions with age, children from all age groups made animate attributions.

Furthermore, an Age by Condition interaction showed that 5-year-olds were impacted by condition, making significantly more animate statements in the Animate Condition. Seven-year-olds and 9-year-olds were not significantly affected by condition. While 7- and 9-year-olds readily used animate terms regardless of condition, 5-year-olds only used animate terms 30% of the time in the neutral condition. This suggests that priming may have a particularly strong effect on younger children. It is possible that young children perceive the video to be animate, but their verbal descriptions might not focus on animacy unless they are specifically prompted. Research has shown that newborn infants are sensitive to animate motion (Di Giorgio et al., 2017; Mascalzoni et al., 2013; Valenza et al., 1996). Therefore, we predicted that the 5-year-old children in our study are aware of the animate nature of the video. If they are not explicitly asked to describe the animate motion, they may focus on other aspects such as shape or color. Older children (7- and 9-year-olds) may be more sensitive to demand characteristics of the task. They may assume that the experimenter wants them to describe the animate interaction without needing to be primed. In contrast, younger children (5-year-olds) may be unable to guess what the experimenter wants to hear and therefore only give animate descriptions when primed.

However, it is important to note that it is also possible that 5-year-old children in the neutral condition did not readily pick up on the animate motion in the videos. Children of this age may have needed the priming cue of animate instructions to interpret the videos as animate. Based on our data, we cannot discern whether 5-year-old children perceived the videos as animate but focused their descriptions on other aspects or did not pick up on animacy cues at all. Various factors such as comfort level in the laboratory, children’s vocabulary, or their understanding of the instructions could have affected performance.

Our results also revealed that Complexity impacted the proportion of animate statements produced by children. When the rectangular figure (house) was present, children made a higher proportion of animate statements about the video. We did not have an a priori prediction of how complexity would impact our results. However, this finding could potentially be explained by the fact that having the house present gives the video context, adding to the storyline and making it easier for them to describe.

Furthermore, we found that the proportion of animate statements used was not impacted by the number of agents present. Again, we did not have an a priori expectation of these variables that might impact our results. Research by Meyerhoff et al. (2013) has shown that increasing the number of moving elements in a display can make chase detection more difficult. However, it is important to note that the number of agents present in our displays did not change the interaction between the dots in the video. The additional agent in videos with three moving dots did not add noise that might distract from the chase. The red dot always chased either one green dot or both green dots in trials with two targets. Therefore, it is unsurprising that adding a third agent to the display did not impact children’s animate attributions.

We also found no significant effect of Outcome (video ending with a catch or no catch). We included the variable as Wagner and Carey (2005) found that even 12-month-old infants expect a chase to end in a catch. We did not have an expectation of how Outcome might affect children’s responses. The lack of an effect of outcome suggests that it was not necessary for the dots to come in contact following the chase for them to be perceived as animate.

Our results showed that age had an effect on the total number of statements made by the child. As age increased, children used more phrases overall to describe what they were seeing. This could be explained in a couple of ways. As children get older, they may feel more comfortable in the laboratory setting and therefore might speak more. It also could be that as children get older, they may simply have a larger vocabulary and therefore may elaborate more.

Overall, our results suggest that school-aged children describe moving shapes in animate terms which is consistent with our original hypothesis. However, these findings differ significantly from the findings of Hu et al. (2010). They found that children rarely make social attributions in their verbal descriptions before the age of nine. Our results revealed that children as young as 5-years-old make social attributions to moving shapes. Our results fit within the literature that provides evidence of young children’s sensitivity toward animacy (Frankenhuis et al., 2013; Poulin-Dubois & Heroux, 1994). One reason our results might differ from those of Hu et al. (2010) is that our measurements of animate attributions differed. Hu et al. showed children videos of cartoon characters twice and then asked children to talk about the content through three separate accounts. The first account was unprompted, but in the second account, the experimenter asked the participants to see the stimuli as characters, implying animacy. In the final account, specific questions were asked about the video. Hu et al. (2010) used multiple instructions for each child and children were only given a combined score across three accounts. Therefore, in Hu et al.’s study, it is hard to tease apart how different instructions may have impacted children’s responses. However, in this study, we are able to directly examine the effect of instructions, as each child only experienced one set of instructions. Another reason that our results may differ is that in this study, the dots were always interacting in the form of a chase. In Hu et al.’s (2010) study, the cartoon characters were shown interacting in various ways. We chose to use chasing motion because previous studies have shown that children are attuned to chasing (Frankenhuis et al., 2013), and therefore, it might be easier for them to interpret that kind of interaction.

In addition, Hu et al. (2010) conducted their study in China, while we conducted our study in Canada. Differences in language as well as cultural differences could potentially influence animacy attributions. For example, parents across various nationalities display distinctly different attitudes and beliefs about parenting (Georgas et al., 2009; Liu & Guo, 2010; Moscardino et al., 2011; Su & Hynie, 2011). Parents in different cultures may have different expectations for their children which may impact how children communicate and verbalize their opinions. Although we do not know for sure how these cultural differences may impact result, it is possible that they do.

### Limitations

We only recruited 5-, 7-, and 9-year-old participants and did not include 6- or 8- year-olds. Our results suggest that there is a developmental change. Without data from 6- and 8-year-olds, our ability to capture whether changes across age happen gradually or rapidly is limited.

Another limitation is that we only used chasing motion. Other types of interactions might yield different results. Future studies should explore children’s verbal descriptions of other types of animate motion such as playing or fighting.

## Conclusion

Overall, this study supports the hypothesis that children, like adults, perceive geometrical figures chasing one another as agents and use animate terms to describe them. Children who were given animate instructions made more animate attributions than children in the Neutral Condition. Older children made more animate statements than younger children. Age and Condition also interacted, such that Condition impacted the proportion of animate phrases used in 5-year-olds but not in 7-year-olds and 9-year-olds. Young children may be more focused on physical aspects of the video such as color and shape of the objects. Priming children with animate instructions may have guided their attention toward the animate nature of the videos and made them disregard other factors. Finally, Complexity was found to impact the proportion of animate phrases used. When the house was present, children used a higher proportion of animate phrases, as it seemed to provide the interaction with more context. Number of Agents and Outcome did not impact the amount of animate attributions in the children’s responses.

## Appendix A

Video 1: One green dot and one red dot moving around the screen. The red dot chases the green dot but no collision or catch occurs.

Video 2: One green dot and one red dot moving around the screen. The red dot chases the green dot and eventually catches it.

Video 3: Two green dots and one red dot moving around the screen. The red dot chases the green dots around the screen. Eventually, the two green dots split up to evade the red chaser, and the red dot does not collide with or catch either green dot.

Video 4: Two green dots and one red dot moving around the screen. The red dot chases the green dots around the screen. Eventually, the two green dots split up to evade the red chaser, but the red dot catches one of them.

Video 5: One green dot and one red dot moving around the screen. The red dot chases the green dot around the screen. The green dot moves into the house to evade the red chaser. The door shuts behind the green dot before the red dot can follow.

Video 6: One green dot and one red dot moving around the screen. The red dot chases the green dot around the screen. The green dot moves into the house to evade the red chaser, but the red dot follows it inside the house and catches it. The door remains open.

Video 7: Two green dots and one red dot moving around the screen. The red dot chases the green dots around the screen. The green dots move into the house to evade the red chaser. The door shuts behind the green dots before the red dot can follow.

Video 8: Two green dots and one red dot moving around the screen. The red dot chases the green dots around the screen. The green dots move into the house to evade the red chaser, but the red dot follows them inside the house and catches one of them. The door remains open.

## Appendix B

Length

0 = no response

1 = for each clause

Coding of responses (score for each clause)

0 = action, non-deliberate (e.g., “moving,” “bouncing,” “rotating,” “a lot of odd movements,” “some shapes spinning around,” “was pushed,” “falling”)

only physical description (e.g., “large,” “color,” “shape”)

1 = use of personal pronouns (he or she)

mention of emotion (e.g., “sad,” “scared,” “tired”)

mention of personality trait (e.g., “mean,” “happy,” “playful,” “aggressive”)

mention of perception (e.g., “sees,” “hears,” “notices”)

deliberate action (e.g., “going,” “ice-skating,” “running,” “jumping”)

deliberate interaction (e.g., “blue and red are fighting,” “child is following their parent,” “red is allowing blue to get close to him”)

mention of mental state (e.g., “red knows,” “red thinks,” “red wants”)

? =undetermined
